# Interplay between Serotonin, Immune Response, and Intestinal Dysbiosis in Inflammatory Bowel Disease

**DOI:** 10.3390/ijms232415632

**Published:** 2022-12-09

**Authors:** Samantha González Delgado, Idalia Garza-Veloz, Fabiola Trejo-Vazquez, Margarita L Martinez-Fierro

**Affiliations:** Molecular Medicine Laboratory, Unidad Académica de Medicina Humana y Ciencias de la Salud, Universidad Autónoma de Zacatecas “Francisco García Salinas”, Zacatecas 98160, Mexico

**Keywords:** Crohn’s disease, ulcerative colitis, serotonin, SERT, *SLC6A4*, inflammation, microbiota, dysbiosis

## Abstract

Inflammatory Bowel Disease (IBD) is a chronic gastrointestinal disorder characterized by periods of activity and remission. IBD includes Crohn’s disease (CD) and ulcerative colitis (UC), and even though IBD has not been considered as a heritable disease, there are genetic variants associated with increased risk for the disease. 5-Hydroxytriptamine (5-HT), or serotonin, exerts a wide range of gastrointestinal effects under both normal and pathological conditions. Furthermore, Serotonin Transporter (SERT) coded by Solute Carrier Family 6 Member 4 (*SLC6A4*) gene (located in the 17q11.1-q12 chromosome), possesses genetic variants, such as Serotonin Transporter Gene Variable Number Tandem Repeat in Intron 2 (STin2-VNTR) and Serotonin-Transporter-linked promoter region (5-HTTLPR), which have an influence over the functionality of SERT in the re-uptake and bioavailability of serotonin. The intestinal microbiota is a crucial actor in normal human gut physiology, exerting effects on serotonin, SERT function, and inflammatory processes. As a consequence of abnormal serotonin signaling and SERT function under these inflammatory processes, the use of selective serotonin re-uptake inhibitors (SSRIs) has been seen to improve disease activity and extraintestinal manifestations, such as depression and anxiety. The aim of this study is to integrate scientific data linking the intestinal microbiota as a regulator of gut serotonin signaling and re-uptake, as well as its role in the pathogenesis of IBD. We performed a narrative review, including a literature search in the PubMed database of both review and original articles (no date restriction), as well as information about the *SLC6A4* gene and its genetic variants obtained from the Ensembl website. Scientific evidence from in vitro, in vivo, and clinical trials regarding the use of selective serotonin reuptake inhibitors as an adjuvant therapy in patients with IBD is also discussed. A total of 194 articles were used between reviews, in vivo, in vitro studies, and clinical trials.

## 1. Introduction

Inflammatory Bowel Disease (IBD) is a clinical term used to refer to Crohn’s disease (CD) and ulcerative colitis (UC), which are chronic intestinal inflammatory pathologies characterized by periods of remission and activity [1]. CD is characterized by its capacity to affect the total gastrointestinal (GI) tract, from the mouth to the anus, whereas UC affects only the colonic mucosa [2]. IBD is particularly prevalent in occidental countries, having the highest prevalence rates in North America and Europe. In Mexico, Yamamoto-Furusho et al. reported 9953 cases of CD and 33,060 cases of UC in 2015 [1]. Numerous studies have been conducted to establish the cause of IBD, including factors such as smoking [3,4], sugary diets [5], drug exposure [6], and oral contraceptives [7], among others [8,9,10,11]. Despite IBD not being considered a hereditary illness, it has been suggested that certain genetic variants may contribute to disease susceptibility, IBD manifestations, and treatment response [12,13]. These genetic variants include p.Arg^702^Trp of *NOD2* (Nucleotide Binding Oligomerization Domain Containing 2) [14], rs11209026 of *IL23R* (IL-23 receptor) [15], rs2241880 of *ATG16L1* (Autophagy-related 16 like 1 protein) [16], and genetic variants found in Serotonin Transporter (SERT) [17,18]. Together with these genetic factors, one of the main mechanisms of injury in IBD is immune-mediated, highlighting the generation of an exaggerated immune response [19,20]. 

**Figure 1:**
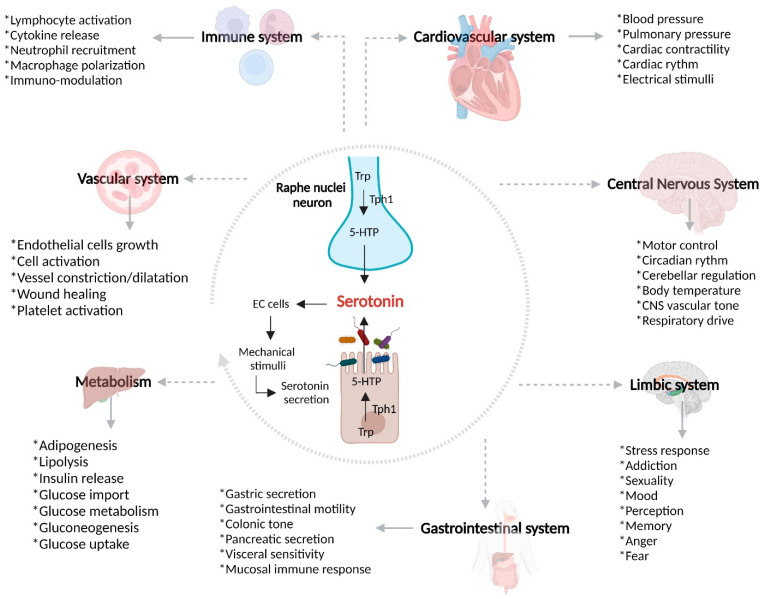
Serotonin biological properties. Serotonin is a well-known monoamine with a wide range of biological properties; it has been seen to be involved in vascular tone and responsible for cardiovascular functions. In the Central Nervous System, it is related to both neurological and cognitive functions such as mood, motor control and others. Interestingly, serotonin is involved in most gastrointestinal functions, from motor to immune properties. Trp, Tryptophan; Tph1, Tryptophan hydroxylase; 5-HTP, 5-hidroxytryptophan; EC, Enterochromaffin cells.

5-Hydroxytryptamine (5-HT), or serotonin, is a monoamine synthesized up to 90% in the GI tract by the enterochromaffin cells (EC) of the GI tract [21], which has been found to be highly related to physiological conditions (Figure 1) and pathogenic GI processes through interactions between serotonin, serotonin receptors, and SERT. 

SERT exerts an important role over the synthesis and clearance of serotonin in organs such as the gut and brain [22]. SERT presents genetic variants which alter its functionality in serotonin re-uptake and bioavailability [23]. The intestinal microbiota plays an important role as a regulator of serotonin bioavailability and immune cell activation [24]. The microbiota is the bacterial, fungal, and viral composition in the GI tract under non-pathological conditions, which plays a critical role in the regulation of neurotransmission. These non-pathogenic micro-organisms maintain the gut epithelial integrity, maturation and modulation of the immune system, and degradation of metabolites such as carbon, polysaccharides, and short-chain fatty acids (SCFAs) [25]. Under normal gut conditions, micro-organisms such as *Bifidobacterium*, *Faecalibacterium prausnitzii*, *Lactobacillus*, *Bacteroides*, *Ruminococcus faecis*, and others have been found; meanwhile, species including *Escherichia coli*, *Shigella flexneri*, *Bilophila wadsorthia*, *Helicobacter pylori*, *Fusobacterium bucleatum*, and *Akkermansia muciniphila* have been found under pathological intestine conditions. This pathological bacterial overgrowth is better known as intestinal dysbiosis [26]. For this review, we aimed to search for scientific evidence linking the intestinal microbiota as a regulator of gut serotonin signaling and re-uptake, as well as its role in immune system over-activation (innate and adaptative) as risk factors for the pathogenesis of IBD. The genetic variants in the SERT gene that may increase the risk of developing IBD and its influence over the functionality of SERT in the re-uptake and bioavailability of serotonin were also integrated in the following sections.

## 2. Structure and Physiology of Gastrointestinal Tract

The intestinal epithelium consists of a wide variety of cells, such as intestinal epithelial cells (IECs), enterochromaffin cells (EC), goblet cells, dendritic cells (DCs), and macrophages, among others [27]. 

Enterocytes comprise one of the main cellular populations. Their main function is the absorption of molecules (e.g., ions, water, vitamins, and biliary acids) in the intestinal lumen, and are also involved in oral tolerance [28,29]. Enterocytes have the capacity to express pattern recognition receptors (PRRs) and pathogen-associated molecular patterns (PAMPs), thus exerting fundamental activities in the immune response [28,30]. Linked to the maintenance of intestinal epithelium integrity, the goblet cells, which are specialized epithelial cells, are essential for the formation of the mucus barrier [31]. These cells are in charge of producing different compounds of the mucus layer, such as mucin (Muc2), with the ability of regulate bacterial adhesion to surface epithelium and intestinal permeability [32]. 

Paneth cells are located at the bottom of Lieberkühn crypts. These cells contain dense granules which store AMP, proteins, cytokines, and proteases [33], and are known for being the main source of AMP, which protects against external stressors and maintains the integrity of the intestinal microbiota [34].

As part of the composition of the neuroendocrine system in the GI tract, the EC (a sub-type of enteroendocrine GI cells) are able to produce close to 95% of serotonin in the organism under mechanical stimuli [35,36]. 

IECs act as a bridge between the microbiota and immune cells through the interaction of cellular unions such as tight junctions (TJs) and antimicrobial peptides (AMPs) [37]. These AMPs have been found to be involved in the immune response and antigen presentation, thanks to the release of cytokines and chemokines [38]. 

DCs are antigen-presenting cells (APC), which are key players in the immune response, acting as a bridge between the innate and adaptative immune response [39]. Gut lamina propria DCs identify antigens and activate a tolerogenic immune response; once they have migrated to mesenteric lymph nodes (MLNs), they present these antigens to T lymphocytes [40]. In the presence of harmful bacteria, intestinal DCs release pro-inflammatory cytokines, such as transforming growth factor β (TGF-β), interleukin 10 (IL-10), and interleukin 6 (IL-6), which are involved in the differentiation of T-naïve lymphocytes to Th2 lymphocytes, while, on the other hand, suppressing Th1 differentiation [41]. 

The wide variety of functions of the GI tract also are controlled in part by the gut–brain axis (GBA) and, in particular, by neurotransmitters such as serotonin [42,43].

## 3. Interplay between Microbiota and Gastrointestinal Tract

The host intestinal microbiota species cohabitate and maintain the intestinal microenvironment. Specially, the microbiota interacts with IECs and immune cells, maintains intestinal barrier integrity, inhibits pathogens on the gut luminal surface, and promotes immune system functioning and the degradation of metabolites such as carbon, polysaccharides, SCFAs, and neurotransmitters to maintain an equilibrium [25]. The microbiota has a wide range of biological properties, one of them being the ability to interact with cell sub-populations such as immune cells, with T lymphocytes and DCs being of special interest [44].

**Figure 2:**
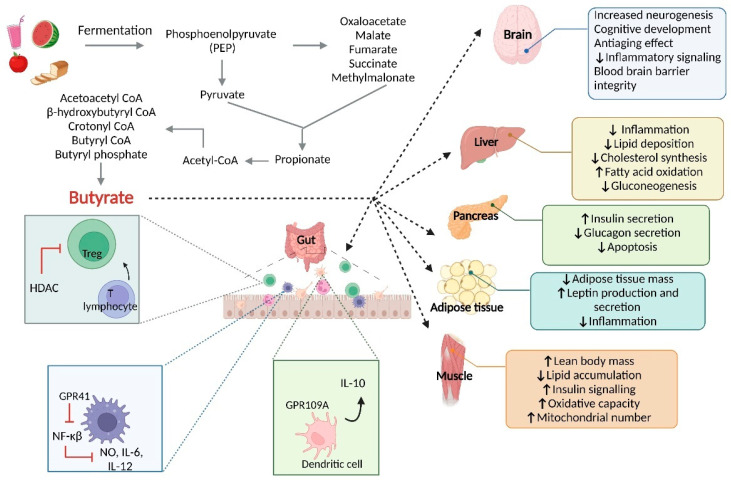
Butyrate synthesis and biological effects. Carbohydrates contained in our daily intake pass through a fermentation process, ending in butyrate production. Butyrate is able to exert various biological effects over a wide range of organs in the organism. Specifically, in the gut, butyrate is able to control the immune response and maintain a tolerogenic profile. IL-10, interleukin 10; IL-6, interleukin 6; NO, nitric oxide; IL-12, interleukin 12; NF-κβ, Nuclear Factor κβ; HDAC, histone deacetylase.

SCFAs produced by the microbiota are the primary products of fermentation of carbohydrates, which can act as energy sources for cell growth and for mucin production [45,46]. SCFAs follow a passive diffusion process to pass across cell membranes, being absorbed by monocarboxylate transporters [47]. Butyrate is the main source of energy used by IECs as a first-line defense by the host in the response to pathogen bacterial invasion (see Figure 2) [48]. 

Butyrate kinase is one of the main enzymes involved in butyrate production [49]. Butyrate has a dual physiologic activity in the colon, where it is able to induce the proliferation of healthy colonocytes and induce apoptosis in damaged cells [46]; furthermore, the role of SCFAs in the regulation of tight junctions (TJs) has been previously described [50]. In the colon, most of the butyrate-producing bacteria are anaerobic, such as *Bifidobacterium*—producers of acetate, fructose, and lactate; bacteria involved in the inhibition of histone deacetylase (HDACs) and activation of G protein-coupled receptors (GPCRs) have been found to be involved in inflammatory processes [47]. The microbiota can exert an influence over the innate and adaptative immune response, with impacts on macrophages and T lymphocytes. Intestinal macrophages enter into the gut to turn into mature macrophages in the gut lamina propria. These mature macrophages have a great phagocytic capacity, executing mechanisms such as NADPH oxidase, ROS, AMPs and proteins [51]. SCFAs also have an influence on macrophage polarization, especially butyrate, which induces M2 macrophage polarization, in turn causing an anti-inflammatory cascade and lower production of ROS [52]. Besides macrophages, the microbiota influences T lymphocytes as the main components of adaptative immunity. SCFAs, such as propionate, have shown a capacity to induce the differentiation of T lymphocytes to Th1 lymphocytes, causing a low inflammatory profile [53]. Additionally, the microbiota may have an influence on neurotransmitter secretion and regulation of the gut–brain axis. 

## 4. Gut–Brain Axis 

**Figure 3:**
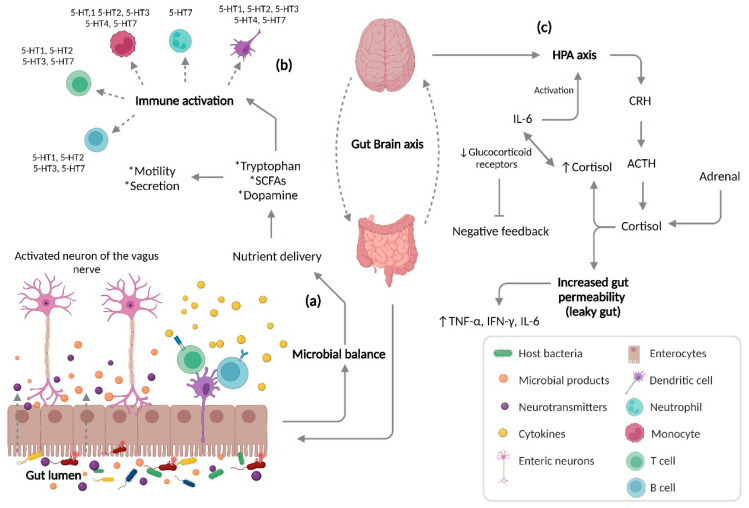
Gut–brain axis. Bidirectional communication between the brain and gut involves a microbial balance (**a**), a phenomenon responsible for producing nutrients such as tryptophan, SCFAs, and dopamine. The biological roles of this nutrient delivery include the activation of enteric neurons in the gut, which control gut homeostasis through functions such as motility and gastrointestinal secretion. In conjunction with gastrointestinal homeostasis, activation of the innate and adaptative immune system (**b**) occurs through signaling of the nutrient delivery from the host bacteria and consequent activation of specific serotonin receptors (e.g., 5-HT1, 5-HT2, 5-HT3, 5-HT4, 5-HT7). Under pathological conditions (**c**), an increased production of pro-inflammatory cytokines such as IL-6 can be observed, with a high potential of activating the HPA axis and decreasing the bioavailability of glucocorticoid receptors, leading to a blockade and negative feedback. As a consequence, there is increased intestinal permeability, accompanied by uncontrolled entrance of microbial products and over-activation of the immune system due to an increased level of cortisol, resulting in increased production of pro-inflammatory cytokines. SCFAs, short-chain fatty acids; HPA, hypothalamic–pituitary–adrenal axis; CRH, corticotropin-releasing hormone; ACTH, adrenocorticotropic hormone; IL-6, interleukin 6; TNF-α, tumor necrosis alpha; IFN-γ, interferon gamma.

The CNS and GI tract communicate through a bidirectional system, better known as the gut–brain axis (GBA), and involving serotonin as a key neurotransmitter. GBA is a complex network of biochemical signaling that involves the CNS and enteric nervous system (ENS; see Figure 3), offering the capacity to execute and regulate normal gut functioning, as well as cognitive and neurodegenerative disorders [54], due to the capacity of the microbiota to generate neurotransmitters and neuromodulators such as tryptophan and glutamine. Under intestinal dysbiosis conditions, there is a possibility that abnormalities can develop in these neuromodulators. A study carried out in germ-free mice indicated decreased levels of tryptophan, tyrosine, and glutamine in their brains [55,56]. In particular, serotonin is one of the main regulators of this axis, affecting various health- and sickness-related processes. 

Serotonin is a neurotransmitter which participates in a wide variety of human biological functions, both in the CNS and in peripheral tissues. Serotonin is synthesized from tryptophan and, therefore, the synthesis of this neurotransmitter depends on the bioavailability of this amino acid, whose catabolism depends on certain specific compounds, such as tryptophan hydroxylase (Tph) and aromatic L-amino acid decarboxylase (AADC) [57]. Tryptophan is an essential amino acid, which means it cannot be produced by the organism. This amino acid is metabolized to serotonin in the brain by the raphe nucleus [58]. Close to 90–95% of serotonin biosynthesis occurs in EC cells [58]. Once synthesized, the release of serotonin from the EC cells occurs due to mechanical stimuli, mainly induced by the alimentary bolus against the intestinal wall; these stimuli result in the activation of mucosal EC cells and submucosal mechanosensitive neurons with motor properties [59]. The release of neurotransmitters such as dopamine, serotonin, and acetylcholine in the gut stimulates afferent primary intrinsic neurons, as well as neurons of the vagal, pelvic, and spinal afferent nerves. This process is controlled by the bacterial production of neurotransmitters such as gamma aminobutyric acid (GABA), serotonin, dopamine, and norepinephrine, produced by bacteria such as *Bifidobacterium*, *Lactobacillus acidophilus*, *Enterococcus*, *Escherichia Coli*, and *Streptococcus* [55,60].

Abnormal proliferation of pathogenic bacteria such as *E. Coli* can cause a disruption of the signaling pathways of the GBA, leading to an increase in pro-inflammatory cytokine levels and serotonin release, and consequently a higher sensitivity of visceral afferent nerves, abnormalities in intestinal permeability, and in GI motility, leading to future GI-affective clinical manifestations [61].

The GBA can exert an influence over the hypothalamic–pituitary–adrenal axis (HPA), leading to activation of the HPA (Figure 3). Certain external stressors, such as stress, pro-inflammatory cytokines (e.g., IL-6), enteropathogenic bacteria, and so on, can induce the release of corticotropin-releasing hormone (CRH), leading to an alteration of the HPA with digestive and neurological manifestations [62]. The GBA, together with the HPA, have been related to autoimmune, chronic, and inflammatory pathologies, as well as neurodegenerative and cognition processes such as Alzheimer’s disease [63], fibromyalgia [64], depression [65], anxiety [66], IBD [67], irritable bowel syndrome (IBS) [68], and others. As a result, under pathological conditions and abnormal GBA and HPA signaling, patients may present increased levels of serotonin, thus favoring different illnesses, especially inflammatory gut processes such as IBD [69].

### 4.1. Serotonin Receptors and Serotonin Transporter (SERT) 

The great amount of 5-HT in the GI system plays a central role in the regulation of processes such as GI secretion, peristalsis, and vasoconstriction, among others [70]. Serotonin exerts powerful and diverse effects on neuronal and other cells through a large family of receptors, consisting of seven distinct classes (i.e., 5-HT1 to 5-HT7). These various serotonin receptors facilitate mechanical and sensitive signaling in the gut. 

#### 4.1.1. 5-HT1 Receptors 

The 5-HT1 receptor and its sub-types (5-HT1A, 5-HT1B, 5-HT1D, 5-HT1E, 5-HT1F, and 5-HT1P; Figure 2) are located in enteric neurons, smooth muscle, Cajal cells, and enterocytes, and exert an effect on the relaxation of the gastric fundus due to prokinetic intestinal stimuli and contraction of the longitudinal and circular muscle layer, as well as secretory and peristaltic reflexes in a serotonin-bioavailability-dependent manner [71,72].

#### 4.1.2. 5-HT2 Receptors

The 5-HT2 receptor and its sub-types (5-HT2A, 5-HT2B, and 5-HT2C) are located in enteric neurons, smooth muscle, enterocytes, Cajal interstitial cells, and connective tissue. These receptors have the capacity to induce contraction of the gastric fundus, as well as relaxation of the longitudinal muscle layer in the gut [72]. The importance of 5-HT2 receptors (specifically 5-HT2B) in the development of ENS has been previously described [73]. As a part of colonic motility, there exists a colonic motor migratory complex (CMMC), which operates to generate rhythmic propulsive contraction through the colon [74].

#### 4.1.3. 5-HT3 Receptors

The 5-HT3 receptor and its sub-type (5-HT3 and 5-HT3A) are ligand-controlled ionic channels, located in enteric neurons, Cajal interstitial cells, enterocytes, extrinsic nerves, and EC cells. This receptor can act in hydrochloric acid secretion and 5-HT liberation by EC cells, as well as increasing intestinal motility [72]. It can be found in mucosa cells and submucosa neuronal bodies, thus regulating autonomous mechanisms such as motility, peristalsis, secretion, and visceral sensibility [75]. It has been reported to be present in cells of the immune system, including DCs, T cells, and B cells, as well as having an influence over the peritoneal macrophages and the subsequent production of pro-inflammatory cytokines, thus promoting the chemotaxis of monocytes to the inflammation area [76].

#### 4.1.4. 5-HT4 Receptors

Finally, 5-HT4 receptors are expressed in central and enteric neurons and EC cells, as well as in monocytes, M cells, and DCs. The activation of these receptors is related to excitatory responses and neuronal post-synaptic potentials [76,77]. In the GI tract, they can accelerate intestinal motility through increasing the release of acetylcholine from the pre-synaptic membrane. In EC cells, they induce the liberation of serotonin [78]. 

The previously mentioned components are responsible for preserving gut homeostasis; under certain external conditions (e.g., infection, inflammation, immune dysregulation, and/or stress), there is a disbalance in these components, consequently leading to the development of susceptibility to GI problems such as IBD [79].

Serotonin cannot cross the membrane of the post-synaptic neurons by an active transport process due to its positive charge; therefore, serotonin clearance requires the activity of SERT, which belongs to the sodium symporters family (NSS) [80]. 

#### 4.1.5. Serotonin Transporter (SERT)

SERT is coded by the Solute Carrier Family 6 Member 4 (*SLC6A4*) gene located in the 17q11.1-q12 chromosome, with a size of 31 kb and 14 exons, coding a 630-amino-acid protein [81]. SERT is expressed in tissues throughout the body, including the heart, blood vessels, platelets, liver, gall bladder, adrenal gland, kidney, immune system, lungs, serotoninergic neurons [82], and in the apical and basolateral membrane of enterocytes [83]. Serotonin can cross cell membranes through a diffusion-like process in different tissues, such as kidney cells, heart cells, endothelial cells, neuronal synaptosomes, and neurons [84,85,86]. As part of the SERT promoter, several single nucleotide genetic variants (SNVs) have influence over serotonin signaling in processes affecting traits such as personality, cognition, and exploratory behavior (Table 1) [87]. 

**Figure 4:**
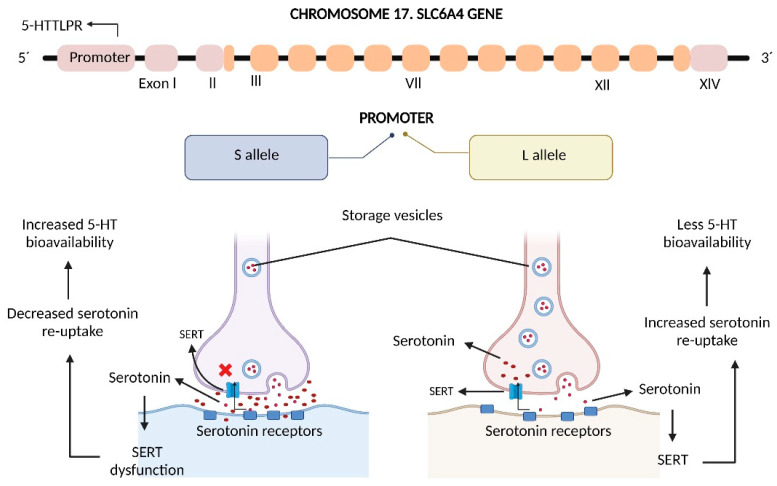
*SLC6A4* gene and 5-HTTLPR act as regulators of serotonergic neurotransmission. The *SLC6A4* gene possesses the 5-HTTLPR genetic variant with two alleles: A short allele (S; 486 bp) and a long allele (L; 530 bp). Both alleles have an influence on SERT functionality, where the S allele is related to lower serotonin re-uptake, leading to increased serotonin bioavailability, while the L allele endows pre-disposition to increased re-uptake by SERT. SERT, Serotonin Transporter; 5HT, 5-hydroxytryptamine.

The serotonin-transporter-linked promoter region (5-HTTLPR) has been closely related with gut pathologies such as IBD and irritable bowel syndrome (IBS). 5-HTTLPR is a region in which an insertion/deletion of 44 bp has been described, comprising a short allele (S) and a long allele (L). The S allele has been related with decreased SERT activity, while the L allele increases SERT expression and serotonin re-uptake [22,106] (Figure 4). In patients with stablished UC diagnosis, higher serotonin concentrations have been observed in those with the L/S genotype [94]; furthermore, a higher prevalence of L/L (39.6%) and L/S (46.9%) genotypes has been observed in patients with CD [92]. Dana Goldner et al. have shown that the S/S genotype led to the highest serotonin levels, and also that those patients with UC in remission had lower frequency of S/S genotype versus controls [107]. Furthermore, extra-long (XL, 17–24 repeats) and extra-short (XS, 11–13 repeats) genotypes have been described specifically in Asian and African populations [108].

## 5. Epidemiology and Pathogenesis of Inflammatory Bowel Disease

UC and CD have been recognized as occidentalized diseases, due to their higher rates of incidence and prevalence in occidental countries [109]. CD mostly presents between 20 and 30 years, whereas UC mostly presents between 30 and 40 years, as well as from 60 to 70 years [19]. 

### 5.1. Risk Factors and Clinical Manifestations

UC and CD have been considered as multi-factorial pathologies, including factors such as antibiotic use, viral and bacterial infections, processed and sugary foods, and alteration in the intestinal microbiota (intestinal dysbiosis) which have been related to an increased risk of IBD [8,110,111,112]. Notably, smoking has been described as having a controversial effect on IBD, as it acts as a risk factor in CD due to the production of free radicals, thus perpetuating inflammation; meanwhile, in UC, smoking has been identified as playing a protective role [3,8]. IBD is associated with factors such as abdominal delivery (CD, OR = 1.38, 95% CI: 1.12–1.70; UC, OR = 1.08, 95% CI: 0.87–1.33), antibiotics exposure (CD, OR = 1.74, 95% CI: 1.35–2.23; UC, OR = 1.08, 95% CI: 0.91–1.27), and sucrose ingestion (CD, RR = 1.09, 95% CI: 1.02–1.16; UC, RR = 1.10, 95% CI: 1.02–1.18), among others [8]. The dietary composition has been also considered as a risk factor, as it has the capacity to disrupt the normal gut microbiota, especially when foods such as sodas, chocolate, and artificial sweeteners are included [5]. Intestinal permeability has been found to be increased in mice fed a high-sugar diet [113]. Besides the influence of diet, increased serum LPS levels and decreased microbiota diversity can lead to reduced production of SCFAs [114]. 

CD is characterized by transmural damage. Patients may present with perianal pain, bleeding, incontinence, fistulization, abscesses, and hemorrhoidal illness [115]. CD can also be characterized by the presence of extraintestinal manifestations, the most common being enthesitis and axial or peripheric arthritis [116,117]. On the other hand, UC can produce chronic inflammation of the colonic mucosa, leading to manifestations such as proctitis, bloody stools, abdominal pain, fatigue, fecal incontinence, arthralgias, and erythema nodosum [118]. In both UC and CD, there is also involvement of the CNS, specifically leading to psychological or psychiatric manifestations [119]. It has been observed that between 15% and 25% of patients with IBD developed depression, while 30% presented with anxiety [120]. Furthermore, it may be accompanied by sleep difficulties and fatigue [121]. As part of the neurological involvement in patients with IBD, deficits in attention and executive function in adults have been observed [122]. 

#### Genetic Susceptibility and Inflammatory Bowel Disease

IBD, similarly to other inflammatory diseases such as autoimmune diseases, has been related to different genes; although IBD has not been recognized as a hereditary disease, several articles provide information about increased susceptibility, disease activity and treatment response related with these genes and their genetic variants (Table 2). As an example, in a trans-ancestry association study, in European, East Asian, Indian and Iranian populations, several risk loci for IBD were found. These loci included *NOD2*, *ATG16L1*, and *IL-23R* [123]. *NOD2* gene variants such as p.Arg^702^Trp were able to provide an increased risk of IBD [14,124]. The rs2241880 gene variant of *ATG16L1* has also been closely related with the maintenance of human intestinal cell homeostasis and autophagy processes in patients with IBD [16,125]. In an interesting review by Nour Younis et al., a wide range of genes related to IBD, together with their reported genetic variants, were considered [13]. Based on wide-genome studies, gene variants in genes such as *ATG16L1* (rs2241880: OR = 0.74; 95% CI: 0.65–0.84; *p* < 0.001), *PTPN2* (Protein tyrosine phosphatase non-receptor type 2) and of *IL-23R* (rs11209026 allele A; OR = 0.32; 95% CI: 0.17–0.60; *p* < 0.001) were related to increased susceptibility to IBD, and even to disease course and treatment outcomes [13]. 

### 5.2. Intestinal Barrier Disruption and Over-Activated Immune Response in Inflammatory Bowel Disease

**Figure 5:**
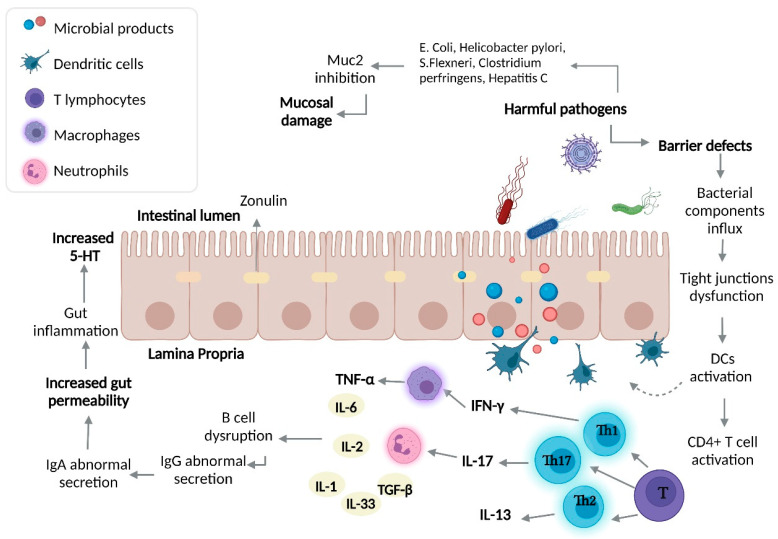
Intestinal dysbiosis and immune response in IBD. The over-growth of harmful bacteria and gut barrier defects cause an influx of bacterial components, leading to activation of the immune response (both innate and adaptative). In UC, Th2 differentiation prevails, while in CD, a Th1 and Th17 adaptative immune response prevails. The influx of bacterial components, adaptative immune response, and harmful bacterial over-growth lead to increased gut permeability, resulting in increased gut 5-HT causing an inflammatory process. DCs, dendritic cells; IFN-γ, interferon-γ; IL-17, interleukin 17; IL-13, interleukin 13; TNF-α, tumor-necrosis factor α.

The intestinal epithelial barrier, together with the intestinal microbiota, are considered an elemental functional unit in the physiology and pathophysiology of the GI. As part of the events that contribute to the pathogenesis of IBD, an alteration in the structure of the intestinal barrier can lead to an altered immune response and intestinal dysbiosis. This phenomenon has been clearly established in pathogen-free mice, where an alteration in the intestinal epithelial cells was observed, together with several abnormalities in the microvilli and a decrease in cellular renovation of the gut (Figure 5) [135]. TJs, such as zonula occludens 1 (ZO-1) and zonula occludens 2 (ZO-2), can be influenced and affected by the intestinal microbiota. TJs can be regulated by bacteria, such as *Lactobacillus rhamnosus*, *Acidophilus plantarum*, and *Bifidobacterium infantis*, through the activation of TLRs, causing increases in the expression of claudin 3, ZO-1, and claudin 4 [136]. In patients with a previous established diagnosis of IBD, an increased paracellular permeability has been observed in almost 40% of patients with CD [137].

Maintenance of the intestinal epithelial integrity depends on the mucus layer; nevertheless, *Akkermansia muciniphila*, *Ruminococcus* spp., *Enterococcus*, *Bifidobacterium* spp., and Bacteroides can degrade mucin and favor the colonization of harmful bacteria [138]. In patients with IBD, increases in *Ruminococcus gnavus*, *Rumminococcus torques*, *Bacteroides fragilis*, and *Bacteroides vulgatus*, containing mucolytic enzymes such as α-galactosidase, sulphatase, neuraminidase, and β-galactosidase, have been observed [139]. An alteration in the relation between *Bacteroidetes*, *Firmicutes*, and *Actinobacteria*, as well as a decrease in *Proteobacteria* and an increase in new bacterial groups, promotes an alteration to homeostasis in a process called intestinal dysbiosis. The equilibrium between host micro-organisms (also called symbiosis) can be affected by nutritive factors, fats, carbohydrates, and drug or antibiotic abuse, among others [110]. Bacteria such as *E. Coli*, *Klebsiella* spp., *Proteus*, *Enterobacter*, *Shigella* spp., *Salmonella* spp., and *Serratia* have been studied as pathogen micro-organisms which are capable of inducing inflammation and intestinal manifestations [135,136].

A decrease has been observed in the fecal concentrations of *Bacteroides fragilis* and *B. vulgatus*, both of which have protective potential, where their absence could lead to perpetuated inflammation and the development of IBD [137]. Reductions in the levels of *Firmicutes* and *Proteobacteria* were found to be the most reported and consistent changes in patients with IBD. Meanwhile, a metagenomic analysis reported an increase in enterobacteria, most commonly *E. coli* [136,138,140]. *F. prausnitzii* possesses anti-inflammatory properties; however, it has been shown to be decreased in patients with IBD (specifically, CD) [141,142]. Some probiotics are able to reinforce the intestinal barrier through the production of defensins and zonula occludens 2 proteins [143]. On the other hand, in patients with IBD, an increase in *Malassezia restricta*—a fungus generally found in the skin, which is able to promote the production of pro-inflammatory cytokines by immune cells—has been observed, specifically in those who were described as having a mutation in the *CARD9* gene, which has been described in IBD [144].

IBD patients with over-expression of carcinoembryonic antigen cell adhesion molecule 6 (CEACAM 6) are more susceptible to EIEC infection, due to the ability of E. coli to bind to CEACAM6 [136]. EIEC has the potential to promote the production of TNF-α by macrophages and survive inside them, thanks to genes such as *ATG16L1*, immunity-related GTPase family protein (IRGM), and *NOD2*. When these genes suffer some mutation, these capacities disappear and the EIEC proteins (FimH) are able to bind to TLR4, generating, as a consequence, an inflammatory response [137]. Furthermore, a relation between serotonin, SERT, and TLR-2 has been observed: Ahmad Qasem et al. found that, after infecting Caco-2 cells with *Mycobacteria paratuberculosis* (MAP), there was an increase in the levels of pro-inflammatory cytokines and TLR2 and, consequently, due to the stimulation of this pro-inflammatory cascade, decreased SERT and IL-10 expression [145]. 

Due to the linkage between intestinal dysbiosis and GBA, several in vitro and in vivo models have demonstrated the important influence of certain pathogenic bacteria present in patients with IBD and/or the beneficial effect of specific bacteria over SERT and serotonin signaling, as detailed in Table 3.

#### 5.2.1. Immune Over-Activation in IBD

Innate lymphoid cells (ILCs) come from a common lymphoid progenitor [155] and are differentiated into Natural Killer cells (NK), innate lymphoid cells 1 (ILC1s), innate lymphoid cells 2 (ILC2s), and innate lymphoid cells 3 (ILC3s) [143]. When ILC1s receive stimuli through IL-12, IL-15, and IL-18, there is a release of interferon gamma (IFN-γ), which promotes the ability of macrophages and DCs to remove intracellular bacteria-presenting antigens through the expression of major histocompatibility complex (MHC) and adhesion molecules. In contrast to ILC1s, ILC2s have the ability to release IL-5, IL-9, and IL-13 under certain stimuli, while ILC3s are producers of IL-22 and IL-17 [140]. An alteration in ILC1s and ILC3s in IBD has been described, as IL-12 is able to induce the differentiation of ILC3s into ILC1S to produce IFN-γ; furthermore, ILC3s can pass into a process of maturation when interacting with the microbiota. Pathogen-free mice with decreased IL-22 level presented with alterations, which led to disruption of the intestinal symbiosis [156]. Furthermore, the interaction of IFN-γ with ILCs promotes the migration of neutrophils, lymphocytes, and macrophages, as well as the activation of endothelial cells, thus causing a disruption in the intestinal barrier by affecting TJs [138]. 

Besides the importance of ILCs, cells such as macrophages are able to act as a bridge between the innate and adaptative immune response. Macrophages are susceptible to a process called polarization, which allows for the differentiation of macrophages into M1 and M2 sub-types, depending on the received stimuli. M1 macrophages are able to trigger an inflammatory response through the production of pro-inflammatory biomarkers such as IL6, IL-12, and TNF-α, while M2 macrophages possess anti-inflammatory properties [139]. In patients with IBD, an increase in the levels of IL-33 and hyperplasia of caliciform cells has been observed, accompanied by macrophage M2 polarization [157]. In particular, in macrophages of patients with IBD, the intracellular replication of bacteria including *E. coli*, *Micobacterium*, *Salmonella*, *Shigella*, *Coxiella*, *Brucella*, *Legionella*, and *Listeria* has been reported [158]. 

Together with the macrophages, neutrophils are the most abundant innate immune cell (approximately 70%). In IBD, these cells are responsible for the increased production of ROS, which causes damage to the intestinal epithelial barrier and can activate an inflammatory cascade [127]. Neutrophils are capable of forming a special defense mechanism—Neutrophil Extracellular Traps (NETs)—which are responsible for catching the pathogen component in a microbicidal environment, guaranteeing regulation of the immune response and a highly efficient defense mechanism [159]. Neutrophils migrate to the area of inflammation through interaction with components such as selectins and intracellular/vascular adhesion molecules (e.g., ICAM-1 and VCAM-1) [160]. One of the main findings in histopathological samples of patients with IBD was neutrophil infiltration with the presence of citrullinated histone H3 (citH3) and some other specific NETs products; this formation of NETs in patients with IBD can be considered a result of release and stimulation by TNF-α [161]. 

Together with macrophages and neutrophils, DCs play an important role in maintaining immune tolerance, considering the effects of nutrients and commensal bacteria [162]. In mucosal samples of patients with IBD, a decrease in sub-populations of CD103^+^ has been observed, as well as an increase in the expression of TLRs, thus generating an increase in immune responses and leading to a loss of immune tolerance [163]. In CD, a disbalance has been documented in the DCs, which may contribute to an excessive T cell response; in samples from patients with CD, a high expression of TLRs (TLR4) was also observed, as well as an increase in CD11c^+^, which produces IL-12 and IL-16 [164].

Once the innate immune response is over, the adaptative immunity is activated. This type of immunity is characterized by the generation of memory, with the capacity to confer long-term immunity, mediated by T and B lymphocytes [165]. 

##### Th1 Response and Crohn’s Disease

An interplay between the microbiota, immune system, and IBD has been described in terms of decreases in *Bacteroides* and *Firmicutes*, along with increases in *Clostridium*, *Gammaproteobacteria*, *Actinobacteria*, enteroinvasive *Escherichia coli* (EIEC), and ILC1s, with high expression of IL-17A, IL-22, and IL-23 receptor (IL-23R) [166]. 

CD4 T helper lymphocytes differentiate to Th1, Th2, Treg, Th17, TFH, or Th9 under specific stimuli; notably, a Th1 and Th17 response has been observed as part of the pathophysiology of CD [166]. As previously mentioned, T lymphocytes can differentiate under various chemical stimuli into Th1 lymphocytes, which are producers of IFN-γ, IL-12, IL-17, and IFN-γ [167]. The IL-17/IL-23 axis is a key actor in CD: when IL-23 binds to its receptor, IL-23R, which is expressed in cells including DCs, macrophages, neutrophils, NK cells and ILCs [168], the activation of a kinase (jak2) and a tirocinkinase (tyk2) causes phosphorylation of the receptor and the transcription 3 activator (STAT3) [169]. Single nucleotide variants (SNVs) reported in the *IL-23R* gene in chromosome 1p31 (rs10889677) lead to increased IL-23R level, favoring chronic inflammation in CD [168]. Furthermore, there exists a relationship between IL-23 and Th17 activation, with subsequent accumulation of IL-17 producer cells in patients with CD [169]. 

##### Th2 Response and Ulcerative Colitis

T lymphocytes differentiate to Th2 lymphocytes after stimulation by IL-4, IL-33, and a transcription factor (GATA3), leading to the final production of IL-4 and IL-13 [170]. Another effect that has been related to IL-13 is the damage that it can impose upon the intestinal epithelial barrier, as IL-13 is able to increase apoptosis in epithelial cells as well as induce disruptions in cellular unions such as claudins-2 [171]. It has been suggested that IL-13 is capable of activating a pro-apoptotic molecule—caspase 3 [172].

### 5.3. Serotonin and Gut–Brain Axis Dysfunction in IBD

Due to the link between intestinal dysbiosis and GBA, it has been considered that there is an important influence of certain pathogenic bacteria present in patients with IBD and/or a beneficial effect of specific bacteria over SERT and serotonin signaling, as detailed in Table 2.

**Figure 6:**
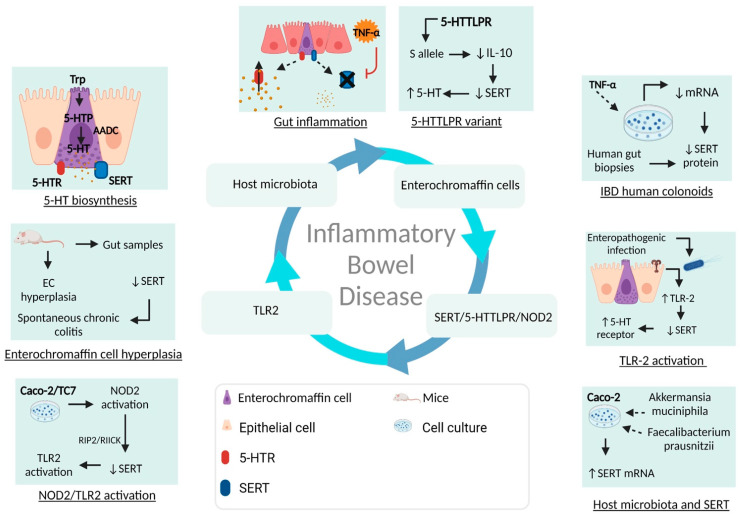
Overview of SERT, gut microbiota, and IBD pathogenesis. Serotonin is a monoamine exerting a wide range of biological effects through the intestinal microbiota, serotonin receptors, and SERT. After the biosynthesis of serotonin in enterochromaffin cells, there is the possibility that, in the context of gut inflammation, SERT may be affected by different pro-inflammatory mediators and bacteria, leading to dysregulation of mRNA, SERT proteins, and serotonin bioavailability. Trp, tryptophan; 5-HTP, 5-hydroxytryptophan; NOD2, Nucleotide oligomerization domain 2; TLR-2, Toll-like receptor 2; EC, enterochromaffin cells.

The GBA coordinates the release of the adrenocorticotropic hormone under stress, which can cause increases in intestinal permeability and glucocorticoid secretion [173]. The increased intestinal permeability associated with a high level of stress favors communication between the microbiota and nervous system [174,175]. Being a complex network, it has an influence over the neuroplasticity of the ENS during inflammation, leading to structural changes, including degradation and loss of enteric ganglion cells, causing an alteration in the normal neurotransmission and, therefore, gastrointestinal mechanosensitive alterations [176]. In view of the pro-inflammatory profile described by IL-1β, IL-6, and TNF-α, among others, leading to inhibition of the vagus nerve and activation of HPA axis (Figure 6) [177], S Haub et al. found that gut inflammation and a lack of IL-10 and SERT lead to abnormal serotonin signaling [178]. Besides the influence of serotonin and SERT over the maintenance of homeostasis in the GI tract, SERT acts as a determinant of the maintenance of bone mass in patients with IBD. Interestingly, osteoporosis is frequent in patients with IBD [179]. B. Lavoie et al. have demonstrated that, in mice, a lack of SERT due to induced colitis led to an incredible loss of trabecular bone mass. It has been found that the serotonin secreted by EC cells acts as a negative regulator of bone density through the inhibition of osteoblasts, which is a cell type responsible for producing and remodeling bone mass [180].

## 6. Selective Serotonin Re-uptake Inhibitors in Inflammatory Bowel Disease: Clinical Evidence

To date, there is evidence of the effectiveness of the previously established therapies for UC and CD treatment, including aminosalicylates, corticosteroids, immunomodulators, and biological therapies [181]. Psychiatric and psychological comorbidities, such as depression, have been correlated to a worse quality of life in patients with IBD [182]. Selective serotonin re-uptake inhibitors (SSRIs) are metabolized by the cytochrome P450 in the liver, with higher specificity to act over SERT compared to other antidepressants; however, patients may develop several adverse effects, including nausea, vomiting, insomnia, drowsiness, extrapyramidal symptoms, serotonin syndrome, and QT prolongation [183]. SSRIs have the potential to increase the synaptic 5-HT concentrations once SERT is blocked [184]. Antidepressants have been used in IBD due to their effects on these psychiatric and psychological comorbidities and certain GI manifestations (Table 4) [185]. Even if there are contradicting conclusions regarding the use and effectiveness of antidepressants such as SSRIs within the clinical spectrum of IBD, there is increasing evidence suggesting the use of SSRIs as a treatment for psychological and psychiatric comorbidities may improve quality of life [186]. In patients with IBD, the use of a combined therapy with SSRIs led to reductions in relapse rates and endoscopic activity [187].

## 7. Concluding Remarks

IBD has been considered as a multi-factorial pathology, as antibiotic use, viral and bacterial infections, processed and sugary foods, and intestinal dysbiosis have all been related to an increased risk of development of this disease. Host microbiota species, such as *Faecalibacterium prausnitzii*, are key actors in regulating enteric serotonin neurotransmission through the production of SCFAs, and, in conjunction with the SERT balance in enterocytes and enterochromaffin cells, are responsible for maintaining gut homeostasis. However, conditions such as intestinal dysbiosis and aberrant SERT pathways result in increased IBD susceptibility. Serotonin and SERT have a close relationship with the complex interaction network in IBD, as well as in the homeostasis of the GI tract, through regulation of serotonin bioavailability. It has been shown that genetic variations in the SERT coding gene can cause abnormal SERT expression in the gut endothelium; these genetic variants, such as STin2 VNTR and 5-HTTLPR, have been found to disturb SERT mRNA expression and SERT protein quantity in both in vitro and in vivo studies, thus leading to decreased serotonin re-uptake, increased serotonin bioavailability, and increased pro-inflammatory cytokine production through serotonin-dependent immune activation. As serotonin is an important monoamine which may induce aberrant inflammatory profiles, leading to gut inflammatory processes such as IBD, we consider that particular interest should be given to serotonin, as well as its receptors and transporters, as actionable factors in relevant therapeutic approaches. Understanding the high impact that IBD has on GBA signaling, there are a wide range of benefits of SSRIs, considering their properties relating to inflammatory and psychiatric comorbidities. Despite the information collected in this review, we consider there is still a lack of information related to SERT genetic variants, such as 5-HTTLPR, as a risk factor—together with environmental influences—in the susceptibility to and development of IBD.

## Figures and Tables

**Table 1:** Serotonin Transporter (SERT) genetic variants and their relation with human diseases.

Genetic Variant	Location	Disease	Effect	References
STin2 VNTR	Intron 2	Migraine Tobacco use disorder	Risk (OR = 1.34; 95% CI: 1.09–1.64) Risk (OR = 3.07; 95% CI: 1.41–6.68)	[88,89]
I425V	Transmembrane region 8	Obsessive–compulsive disorder	Risk (OR = 6.54; 95% CI: 1.7–24.8)	[90]
5-HTTLPR	Promoter	Irritable bowel syndrome GTS Crohn’s disease MC and Ulcerative Colitis	Risk (Allele S; OR = 1.36) Risk (S/S; OR = 1.5; 95% CI: 0.8–2.98) Risk (L; OR = 0.9; 95% CI: 0.4–2.0) Higher levels of serotonin (*p* < 0.01) *	[91,92,93,94]
rs3813034	3′ UTR	Panic disorder	Risk (OR = 1.44; 95% CI: 1.13–1.85)	[95]
rs3794808	Intron	Irritable bowel syndrome	No significant association *	[96]
rs140701	Intron	Breast cancer	Risk (OR = 1.56; 95% CI: 1.01–2.41)	[97]
rs4583306	Intron	Obsessive–compulsive symptoms	Relation with cleanliness dimension (*p* = 0.004) *	[98]
rs140700	Intron	Primary insomnia Schizophrenia	Not risk factor (OR = 1.32; 95% CI: 0.49–3.55) No association *	[99,100]
rs2020942	Intron	Migraine	No significant association (OR = 1.09; 95% CI: 0.82–1.44)	[101]
rs12150214	Intron	Colorectal cancer Antidepressant response	Shorter overall survival (OR = 1.57; 95% CI: 1.14–2.16) Poorer response to fluoxetine (OR = 4.24; 95% CI: 1.39–12.98)	[102,103]
rs2066713	Intron	Autism Schizophrenia	No significant association * Significant association (*p* < 0.001)*	[104,105]

* Not reported OR. STin2 VNTR: Serotonin Transporter Gene Variable Number Tandem Repeat in Intron 2; 5-HTTLPR: serotonin-transporter-linked promoter region; GTS: Gilles de la Tourette syndrome; MC: microscopic colitis; 3′ UTR: 3′ untranslated region.

**Table 2:** Genes related to Inflammatory Bowel Disease.

Gene	Locus	Effect	Reference
*NOD2*	16q12.1	IBD increased risk	[13,14,123,126]
*ATG16L1*	2q37.1	Impaired intracellular bacteria clearance in IBD, intestinal autophagy	[13,16,127,128]
*PTPN2*	18p11.21	IBD increased risk	[13,129,130]
*IL-23R*	1p31.3	IBD susceptibility, Crohn’s disease risk	[13,15,131]
*IL-10*	1q32.1	IBD steroid dependency, early onset IBD	[13,132,133]
*HNF4α*	20q13.12	IBD susceptibility	[13,18,134]

NOD2: Nucleotide-binding oligomerization domain-containing protein 2; ATG16L1: Autophagy-related 16-like 1 protein; PTPN2: Protein tyrosine phosphatase non-receptor type 2; IL-23R: Interleukin 23 receptor; IL-10: Interleukin 10; HNF4α: Hepatocyte Nuclear Factor 4 alpha.

**Table 3:** Relationships between bacteria and SERT function.

Bacteria	Mechanism	Model	References
*Enteropathogenic E. coli*	Activation of protein tyrosine phosphatase, a process that leads to SERT inhibition	Caco-2 cells infected with *E. coli*	[146,147]
*Listeria* *monocytogenes*	Reduced SERT expression related to a transcriptional change in TLR10 and TLR2	Caco-2/TC7 cells infected with *Listeria monocytogenes*	[148]
*Akkermansia* *muciniphila*	Interaction between activated TLR2 and SERT causes reduced SERT expression	Caco-2 cells infected with *Akkermansia muciniphila*	[149]
*Lactobacillus* *acidophilus*	Up-regulation of SERT mRNA	*Lactobacillus acidophilus* and *B. longum* interaction with HT-29 and Caco-2 cells	[150]
*Lactobacillus* *rhamnosus*	SERT Gene and protein up-regulation	Wistar rats implemented with probiotics and prebiotics	[151]
*Campylobacter jejuni*	EC hyperplasia and reduced SERT expression	C57BL/6 mice infected with *T. Spiralis* and *C. jejuni*	[152]
*Salmonella* *typhimurium*	Inhibition of SERT by TLR4 activation	Mice and Caco-2 cells infected with *S. typhimurium*	[153,154]

**Table 4:** Scientific evidence regarding the use of selective serotonin re-uptake inhibitors in patients with inflammatory bowel disease.

Type of study	Results	Reference
Review	Antidepressants are highly used for depression and anxiety problems in IBD, even though gut side-effects are questionable	[182]
Retrospective	Antidepressants showed a protective role over IBD	[185]
Review	Useful effects: anti-inflammatory properties, immune regulation	[120]
Retrospective	Increased risk of corticosteroid dependency after long-term SSRI intake	[188]
In vivo	Decreased stool output, delayed transit, and attenuated colonic sensitivity related with paroxetine intake	[189]
Longitudinal	Antidepressants predispose lower medical therapy escalation	[186]
Review	The results for the outcomes are uncertain	[190]
In vitro	Fluoxetine inhibited NF-κβ and up-regulated expression of IL-8 in COLO 205 colon epithelial cells stimulated with TNF-α	[191]
Prospective, randomized, double-blind, and placebo-controlled clinical trial	Venlafaxine reduced TNF-α levels in patients with IBD	[192]
Double-blind	Duloxetine can be used as a therapy for reducing depression, anxiety, and severity of physical symptoms	[193]
Population-based cohort study. Prospectively collected data	Patients with IBD and a 180-day antidepressant therapy showed lower relapse rates, hospitalization, and less risk of initiating anti-TNF therapy	[194]

## Data Availability

Data sharing not applicable to this article as no new datasets were generated or analyzed during the current review.
